# Supplementary material for: Virtual Screening of a Library of Naturally Occurring Anthraquinones for Potential Anti-Fouling Agents
Source: Molecules. 2023 Jan 19;28(3):995. doi: 10.3390/molecules28030995 (PMC9920117; doi:10.3390/molecules28030995)
Supplement: Supplementary file 1 [file molecules-28-00995-s001.zip › molecules-2076841-supplementary.pdf]

**Table S1**: Dataset of 2194 natural AQs-related compounds from COCONUT [55] with docking score			
**S.No.**		**Binding Affinity (kcal/mol)**	**Coconut_id**
1	D	-7.2	CNP0164117
2	D	-7	CNP0287592
3	D	-6.9	CNP0182500
4	D	-7	CNP0283796
5	D	-4.8	CNP0288616
6	D	-4.6	CNP0087351
7	D	-6.6	CNP0122539
8	D	-7.5	CNP0163297
9	D	-7.8	CNP0384131
10	D	-6.2	CNP0071815
11	D	-4.8	CNP0114091
12	D	-6.1	CNP0151804
13	D	-6.1	CNP0071814
14	D	-5.9	CNP0404841
15	D	-6.8	CNP0207329
16	D	-5.9	CNP0296102
17	D	-6	CNP0192641
18	D	-5.1	CNP0353184
19	D	-5	CNP0324808
20	D	-7.6	CNP0298341
21	D	-7.4	CNP0302049
22	D	-7.1	CNP0405524
23	D	-7.3	CNP0232100
24	D	-5.3	CNP0081162
25	D	-6	CNP0191322
26	D	-3.1	CNP0394232
27	D	-5.3	CNP0211140
28	D	-4.7	CNP0131555
29	D	-3.6	CNP0174591
30	D	-1.6	CNP0175904
31	D	-0.4	CNP0344609
32	D	11.9	CNP0392895
33	D	-7.2	CNP0435526
34	D	-5	CNP0359929
35	D	-5.6	CNP0243027
36	D	-2.2	CNP0328628
37	D	7.6	CNP0425426
38	D	21	CNP0227422
39	D	-6.5	CNP0307572
40	D	-7.5	CNP0257097
41	D	-7.8	CNP0112288
42	D	-7.1	CNP0184539
43	D	-7.3	CNP0154507
44	D	-7.3	CNP0267437
45	D	-7.2	CNP0309152
46	D	-5.2	CNP0331306
47	D	-6.2	CNP0218669
48	D	-4.7	CNP0171982
49	D	-6.2	CNP0181824
50	D	-5.9	CNP0164344
51	D	-6.1	CNP0304503
52	D	-9.4	CNP0229787

53	D	-6.5	CNP0357065
54	D	-6.3	CNP0237073
55	D	-6.3	CNP0300529
56	D	-6.8	CNP0404399
57	D	-7.1	CNP0177629
58	D	-7.3	CNP0165712
59	D	-6.8	CNP0263252
60	D	-7.2	CNP0107083
61	D	-7.2	CNP0286267
62	D	-6.8	CNP0142077
63	D	-6.4	CNP0352396
64	D	-6.9	CNP0113376
65	D	-8.1	CNP0219593
66	D	-6.7	CNP0314074
67	D	-7.2	CNP0223881
68	D	-8.1	CNP0327787
69	D	-6.1	CNP0195505
70	D	-	CNP0377899
71	D	-7.8	CNP0101949
72	D	-	CNP0088260
73	D	-6.9	CNP0337939
74	D	-7	CNP0286852
75	D	-7	CNP0287189
76	D	-6.3	CNP0149007
77	D	-6.2	CNP0229375
78	D	-6	CNP0340948
79	D	-7.3	CNP0293601
80	D	-8	CNP0390697
81	D	-6.9	CNP0016843
82	D	-6.2	CNP0125418
83	D	-7.5	CNP0171010
84	D	-7.2	CNP0310182
85	D	-6.4	CNP0259487
86	D	-8.5	CNP0304999
87	D	-6.9	CNP0008370
88	D	-8.6	CNP0339449
89	D	-6.2	CNP0113663
90	D	-6.7	CNP0088960
91	D	-7.8	CNP0328129
92	D	-7.5	CNP0042681
93	D	-7.3	CNP0384010
94	D	-7.8	CNP0006629
95	D	-6.5	CNP0331096
96	D	-6.7	CNP0080924
97	D	-7	CNP0173058
98	D	-7.7	CNP0010229
99	D	-8	CNP0303226
100	D	-9.2	CNP0309114
101	D	-3.9	CNP0008289
102	D	-3.2	CNP0164418
103	D	-4.5	CNP0143467
104	D	-3.9	CNP0167507
105	D	-4.1	CNP0213628
106	D	-3.3	CNP0161477
107	D	-4.3	CNP0140125

108	D	-4.7	CNP0206906
109	D	-	CNP0134328
110	D	-4.7	CNP0093731
111	D	-4	CNP0008907
112	D	-4.3	CNP0227933
113	D	-3.2	CNP0144159
114	D	-4.9	CNP0198387
115	D	-4.3	CNP0307485
116	D	-4.1	CNP0038899
117	D	-4.8	CNP0220359
118	D	-4.4	CNP0099782
119	D	-	CNP0420702
120	D	-5	CNP0139645
121	D	-6.5	CNP0287462
122	D	-10.2	CNP0388629
123	D	-8.3	CNP0190140
124	D	-8	CNP0121057
125	D	-6.7	CNP0026058
126	D	-8.5	CNP0008121
127	D	-9	CNP0384871
128	D	-8.1	CNP0261767
129	D	-6	CNP0259341
130	D	-8.4	CNP0197369
131	D	-	CNP0212758
132	D	-9	CNP0316412
133	D	-7.9	CNP0337510
134	D	-6.7	CNP0123094
135	D	-7.5	CNP0242086
136	D	-6.9	CNP0147512
137	D	-8	CNP0089143
138	D	-8	CNP0130818
139	D	-7	CNP0412379
140	D	-7.6	CNP0397274
141	D	-8	CNP0277276
142	D	-9	CNP0009212
143	D	-8.3	CNP0341411
144	D	-7.5	CNP0008403
145	D	-7.6	CNP0099941
146	D	-9.5	CNP0323042
147	D	-8.3	CNP0363929
148	D	-8.1	CNP0195518
149	D	-7.6	CNP0201432
150	D	-8.3	CNP0329615
151	D	-8.3	CNP0091974
152	D	-7.5	CNP0261708
153	D	-8.3	CNP0343067
154	D	-7.4	CNP0237663
155	D	-9.4	CNP0404006
156	D	-7	CNP0008057
157	D	-6.6	CNP0283504
158	D	-6.8	CNP0090465
159	D	-7.7	CNP0296027
160	D	-7.9	CNP0191074
161	D	-6.4	CNP0215058
162	D	-7.5	CNP0245106

163	D	-6.7	CNP0154260
164	D	-6.3	CNP0418470
165	D	-6.6	CNP0038548
166	D	-6.2	CNP0062835
167	D	-7.8	CNP0179841
168	D	-7.1	CNP0018884
169	D	-6.8	CNP0087849
170	D	-6.4	CNP0115943
171	D	-7.2	CNP0243128
172	D	-8.2	CNP0419182
173	D	-7.6	CNP0121854
174	D	-7.5	CNP0316445
175	D	-6.8	CNP0316724
176	D	-6.4	CNP0152482
177	D	-8	CNP0111804
178	D	-8	CNP0138873
179	D	-6.6	CNP0298296
180	D	-7.5	CNP0285204
181	D	-6.5	CNP0274786
182	D	-7.3	CNP0177353
183	D	-6.2	CNP0232451
184	D	-	CNP0251119
185	D	-7.1	CNP0235018
186	D	-8.5	CNP0266857
187	D	-8.4	CNP0118833
188	D	-8.8	CNP0394381
189	D	-7.4	CNP0346155
190	D	-9	CNP0343733
191	D	-6.1	CNP0340406
192	D	-7.3	CNP0422568
193	D	-8.4	CNP0105186
194	D	-8.4	CNP0006599
195	D	-6.2	CNP0167753
196	D	-8	CNP0421164
197	D	-7	CNP0070907
198	D	-6.7	CNP0120710
199	D	-8.8	CNP0281894
200	D	-7.6	CNP0265474
201	D	-8.2	CNP0391141
202	D	-8.8	CNP0415101
203	D	-8.1	CNP0418451
204	D	-7.2	CNP0370610
205	D	-8	CNP0282759
206	D	-8.3	CNP0196850
207	D	-7.7	CNP0257054
208	D	-	CNP0099320
209	D	-8.1	CNP0474581
210	D	-7.9	CNP0220978
211	D	-6.4	CNP0275801
212	D	-7.6	CNP0347117
213	D	-	CNP0234540
214	D	-7.9	CNP0105524
215	D	-9.4	CNP0203367
216	D	-	CNP0096800
217	D	-6.5	CNP0247064

218	D	-7.5	CNP0398637
219	D	-8.9	CNP0298017
220	D	-8	CNP0172917
221	D	-7.2	CNP0334591
222	D	-7.2	CNP0365115
223	D	-7.4	CNP0172299
224	D	-7.8	CNP0344053
225	D	-8.5	CNP0224978
226	D	-8.1	CNP0176439
227	D	-7.4	CNP0111729
228	D	-6.4	CNP0134819
229	D	-8.4	CNP0258317
230	D	-8.3	CNP0193792
231	D	-6.9	CNP0422268
232	D	-7.4	CNP0319805
233	D	-8.7	CNP0181567
234	D	-7.4	CNP0473868
235	D	-6.6	CNP0098961
236	D	-6.9	CNP0018904
237	D	-6.2	CNP0329181
238	D	-6.1	CNP0291156
239	D	-7.5	CNP0216821
240	D	-7.9	CNP0158617
241	D	-7.4	CNP0148308
242	D	-8.3	CNP0166617
243	D	-8	CNP0090492
244	D	-6.8	CNP0217425
245	D	-6.9	CNP0063925
246	D	-6.3	CNP0285568
247	D	-7.1	CNP0289351
248	D	-6	CNP0297856
249	D	-7	CNP0298869
250	D	-6.1	CNP0093905
251	D	-7.6	CNP0107981
252	D	-6.5	CNP0343684
253	D	-6.5	CNP0279061
254	D	-7.7	CNP0270217
255	D	-7	CNP0353193
256	D	-7.8	CNP0080922
257	D	-7.2	CNP0102378
258	D	-6.4	CNP0271728
259	D	-7.8	CNP0177105
260	D	-7.9	CNP0306519
261	D	-8	CNP0417535
262	D	-8	CNP0230160
263	D	-8.2	CNP0124312
264	D	-9.6	CNP0259479
265	D	-6.4	CNP0341948
266	D	-7.4	CNP0255748
267	D	-8	CNP0013627
268	D	-7.1	CNP0191906
269	D	-6.7	CNP0130852
270	D	-6.9	CNP0172795
271	D	-6.4	CNP0377402
272	D	-6	CNP0177246

273	D	-6.4	CNP0127006
274	D	-8.9	CNP0034140
275	D	-7.5	CNP0088758
276	D	-7.4	CNP0413658
277	D	-6.1	CNP0102842
278	D	-6.3	CNP0382128
279	D	-7.7	CNP0266989
280	D	-7.7	CNP0274104
281	D	-8.6	CNP0417538
282	D	-8.5	CNP0013821
283	D	-7	CNP0291455
284	D	-6.1	CNP0280363
285	D	-6.3	CNP0301984
286	D	-7.9	CNP0080909
287	D	-6.4	CNP0282438
288	D	-6.6	CNP0337316
289	D	-8	CNP0008436
290	D	-7.2	CNP0206008
291	D	-7	CNP0289013
292	D	-7.3	CNP0009754
293	D	-8.1	CNP0115064
294	D	-8.4	CNP0108644
295	D	-7.4	CNP0342334
296	D	-8.1	CNP0295613
297	D	-7.7	CNP0035593
298	D	-9.2	CNP0129460
299	D	-7.1	CNP0230619
300	D	-5.9	CNP0089147
301	D	-8.1	CNP0110759
302	D	-9.5	CNP0097600
303	D	-8.7	CNP0113100
304	D	-8.4	CNP0340799
305	D	-6.9	CNP0209493
306	D	-9	CNP0215855
307	D	-10.2	CNP0408325
308	D	-9.3	CNP0264954
309	D	-6.6	CNP0347456
310	D	-9.4	CNP0019876
311	D	-10.5	CNP0093442
312	D	-6.9	CNP0305503
313	D	-8.3	CNP0109260
314	D	-7.3	CNP0258562
315	D	-7.9	CNP0270127
316	D	-9.1	CNP0301002
317	D	-8.7	CNP0371080
318	D	-8.1	CNP0008067
319	D	-8.1	CNP0139138
320	D	-7.7	CNP0292643
321	D	-7.7	CNP0271984
322	D	-7.2	CNP0072680
323	D	-6.1	CNP0170169
324	D	-9.8	CNP0024629
325	D	-8.9	CNP0091179
326	D	-7.7	CNP0008919
327	D	-8.3	CNP0070857

328	D	-7.6	CNP0120874
329	D	-8.9	CNP0140926
330	D	-6.8	CNP0333885
331	D	-7.3	CNP0378354
332	D	-7.4	CNP0274920
333	D	-8	CNP0272431
334	D	-9.5	CNP0206787
335	D	-8.9	CNP0300624
336	D	-7.5	CNP0284719
337	D	-7.5	CNP0109575
338	D	-8.1	CNP0363606
339	D	-9	CNP0080910
340	D	-7.7	CNP0293406
341	D	-8.9	CNP0000006
342	D	-8.4	CNP0227967
343	D	-7.6	CNP0358079
344	D	-9.4	CNP0391210
345	D	-8.3	CNP0199241
346	D	-6.2	CNP0103980
347	D	-9	CNP0024475
348	D	-9.4	CNP0270904
349	D	-10.4	CNP0246653
350	D	-10.2	CNP0347371
351	D	-7.7	CNP0143216
352	D	-7.4	CNP0091568
353	D	-8.7	CNP0236326
354	D	-7.8	CNP0143462
355	D	-8.2	CNP0298156
356	D	-8.3	CNP0135754
357	D	-6.8	CNP0223272
358	D	-8.2	CNP0333756
359	D	-	CNP0100942
360	D	-8.8	CNP0328905
361	D	-9.1	CNP0090571
362	D	-8.1	CNP0267295
363	D	-7.7	CNP0252780
364	D	-8.4	CNP0008174
365	D	-7.5	CNP0284490
366	D	-7.6	CNP0008401
367	D	-6.7	CNP0399324
368	D	-7.2	CNP0197457
369	D	-7.3	CNP0058331
370	D	-7	CNP0240408
371	D	-6.7	CNP0093515
372	D	-6.2	CNP0121942
373	D	-	CNP0340003
374	D	-7.7	CNP0091170
375	D	-7.4	CNP0313114
376	D	-6.9	CNP0204787
377	D	-7.3	CNP0408825
378	D	-7.9	CNP0338317
379	D	-6.6	CNP0108003
380	D	-7.3	CNP0353624
381	D	-6.6	CNP0262787
382	D	-8.7	CNP0061269

383	D	-7.6	CNP0008056
384	D	-7.4	CNP0307899
385	D	-7.9	CNP0040853
386	D	-10.8	CNP0137516
387	D	-7.1	CNP0310103
388	D	-6.8	CNP0061384
389	D	-7.4	CNP0225057
390	D	-7.5	CNP0223871
391	D	-7.9	CNP0239929
392	D	-7.4	CNP0128474
393	D	-6.9	CNP0117731
394	D	-6.7	CNP0008054
395	D	-6.4	CNP0333869
396	D	-7.9	CNP0088639
397	D	-6.1	CNP0244827
398	D	-	CNP0321061
399	D	-6.5	CNP0362973
400	D	-6.8	CNP0190111
401	D	-7.9	CNP0366303
402	D	-8.5	CNP0087994
403	D	-6.4	CNP0078429
404	D	-6.4	CNP0361371
405	D	-9.1	CNP0088747
406	D	-7.1	CNP0149812
407	D	-7.8	CNP0308261
408	D	-6.8	CNP0309301
409	D	-6.4	CNP0235219
410	D	-6.4	CNP0042161
411	D	-7.2	CNP0197151
412	D	-6.7	CNP0102176
413	D	-8.1	CNP0258374
414	D	-6.6	CNP0006745
415	D	-7.5	CNP0213103
416	D	-7.5	CNP0094342
417	D	-7.2	CNP0362963
418	D	-8.9	CNP0098512
419	D	-7.5	CNP0149635
420	D	-7.3	CNP0157721
421	D	-8.5	CNP0356198
422	D	-7.2	CNP0259724
423	D	-6.6	CNP0118145
424	D	-7	CNP0097469
425	D	-6.3	CNP0164200
426	D	-6.8	CNP0369952
427	D	-6.3	CNP0323611
428	D	-6	CNP0178649
429	D	-9.5	CNP0089496
430	D	-7.5	CNP0319528
431	D	-	CNP0088249
432	D	-6.5	CNP0251865
433	D	-7.7	CNP0263329
434	D	-8	CNP0419781
435	D	-6.5	CNP0318717
436	D	-7	CNP0080250
437	D	-7.5	CNP0008055

438	D	-9.1	CNP0092220
439	D	-7.1	CNP0090018
440	D	-8.2	CNP0395463
441	D	-7.3	CNP0244170
442	D	-8.2	CNP0008422
443	D	-6.7	CNP0132480
444	D	-7.8	CNP0090079
445	D	-7.4	CNP0117152
446	D	-7.2	CNP0263965
447	D	-7.3	CNP0253307
448	D	-7.2	CNP0133593
449	D	-8.2	CNP0323961
450	D	-8.6	CNP0113228
451	D	-6.4	CNP0070350
452	D	-7.2	CNP0141448
453	D	-7.5	CNP0173780
454	D	-6.7	CNP0130857
455	D	-7.5	CNP0418163
456	D	-7.3	CNP0155599
457	D	-7.7	CNP0167283
458	D	-8.5	CNP0008553
459	D	-6.8	CNP0179564
460	D	-7.7	CNP0218715
461	D	-8.1	CNP0054339
462	D	-8.6	CNP0311496
463	D	-9	CNP0092949
464	D	-6.8	CNP0146545
465	D	-9.8	CNP0000012
466	D	-7.7	CNP0243033
467	D	-9.3	CNP0419569
468	D	-7.4	CNP0099898
469	D	-8.1	CNP0246659
470	D	-6.6	CNP0214725
471	D	-8.1	CNP0221309
472	D	-7.4	CNP0235726
473	D	-11.3	CNP0419758
474	D	-7.2	CNP0329393
475	D	-7.1	CNP0000008
476	D	-6.6	CNP0423373
477	D	-6.3	CNP0232869
478	D	-8.6	CNP0388669
479	D	-7	CNP0129388
480	D	-7.1	CNP0266131
481	D	-8.5	CNP0088931
482	D	-8.8	CNP0184132
483	D	-7.9	CNP0092647
484	D	-7.8	CNP0351381
485	D	-6.3	CNP0335585
486	D	-6.9	CNP0340753
487	D	-8.2	CNP0092225
488	D	-7.5	CNP0000132
489	D	-7.6	CNP0322100
490	D	-7.8	CNP0394523
491	D	-7.1	CNP0008173
492	D	-7.5	CNP0391290

493	D	-7.8	CNP0354447
494	D	-7.6	CNP0098508
495	D	-7.9	CNP0008070
496	D	-8	CNP0083167
497	D	-8	CNP0106464
498	D	-8.9	CNP0354121
499	D	-8.5	CNP0009138
500	D	-6.6	CNP0339294
501	D	-6.9	CNP0099005
502	D	-7.1	CNP0115851
503	D	-7.9	CNP0237195
504	D	-	CNP0251071
505	D	-7.1	CNP0087409
506	D	-6.8	CNP0421440
507	D	-8.4	CNP0379931
508	D	-7.6	CNP0305668
509	D	-8.4	CNP0338056
510	D	-7.4	CNP0317839
511	D	-7.6	CNP0133005
512	D	-6.3	CNP0188878
513	D	-7	CNP0330006
514	D	-8.2	CNP0385902
515	D	-8.3	CNP0008893
516	D	-8.3	CNP0281908
517	D	-8.5	CNP0378596
518	D	-7.4	CNP0201573
519	D	-7.2	CNP0358610
520	D	-6.4	CNP0202536
521	D	-8.4	CNP0125178
522	D	-7.7	CNP0169729
523	D	-8.3	CNP0013628
524	D	-	CNP0353259
525	D	-7.4	CNP0273686
526	D	-6.3	CNP0187636
527	D	-7.2	CNP0220267
528	D	-8.4	CNP0417021
529	D	-7.8	CNP0353124
530	D	-7.5	CNP0270375
531	D	-7.7	CNP0134985
532	D	-8.1	CNP0056247
533	D	-6.9	CNP0337328
534	D	-8.8	CNP0311311
535	D	-7.7	CNP0115183
536	D	-7.5	CNP0332835
537	D	-9.3	CNP0102179
538	D	-7.9	CNP0202354
539	D	-9.1	CNP0116100
540	D	-7.5	CNP0031315
541	D	-9.9	CNP0089315
542	D	-6.7	CNP0281779
543	D	-7.8	CNP0171481
544	D	-7.8	CNP0384861
545	D	-7.3	CNP0325219
546	D	-9.1	CNP0306008
547	D	-7.6	CNP0194956

548	D	-7.5	CNP0104897
549	D	-8.1	CNP0025327
550	D	-7	CNP0423058
551	D	-8.9	CNP0113212
552	D	-7.5	CNP0423565
553	D	-8.6	CNP0048999
554	D	-7.2	CNP0161289
555	D	-8.5	CNP0379961
556	D	-7.5	CNP0113949
557	D	-7.7	CNP0008064
558	D	-6.9	CNP0417146
559	D	-7.8	CNP0387617
560	D	-6.7	CNP0180709
561	D	-7.7	CNP0282852
562	D	-6.7	CNP0210814
563	D	-7.3	CNP0418122
564	D	-8.2	CNP0178472
565	D	-7	CNP0196059
566	D	-8.4	CNP0117276
567	D	-8	CNP0423253
568	D	-6.8	CNP0160328
569	D	-7.3	CNP0302117
570	D	-10.2	CNP0356898
571	D	-10.3	CNP0311484
572	D	-9.9	CNP0096821
573	D	-9.8	CNP0174206
574	D	-7	CNP0305468
575	D	-	CNP0088761
576	D	-7.4	CNP0302722
577	D	-7.1	CNP0420416
578	D	-7	CNP0250494
579	D	-7.4	CNP0257839
580	D	-8.4	CNP0166260
581	D	-7.9	CNP0333829
582	D	-8.7	CNP0006762
583	D	-8	CNP0142149
584	D	-8.7	CNP0131658
585	D	-6.3	CNP0109775
586	D	-7.4	CNP0269271
587	D	-6.7	CNP0196995
588	D	-8.6	CNP0369594
589	D	-8.7	CNP0116146
590	D	-6.8	CNP0234180
591	D	-7.2	CNP0276756
592	D	-7.9	CNP0288172
593	D	-6.5	CNP0145046
594	D	-7.1	CNP0286831
595	D	-7.6	CNP0341599
596	D	-7.1	CNP0121135
597	D	-6.3	CNP0148970
598	D	-8.4	CNP0326722
599	D	-6.8	CNP0310831
600	D	-6.2	CNP0351670
601	D	-7.7	CNP0168650
602	D	-6.7	CNP0306330

603	D	-6.6	CNP0300532
604	D	-7.8	CNP0316848
605	D	-7.7	CNP0175015
606	D	-8.2	CNP0000004
607	D	-7.8	CNP0239267
608	D	-6.7	CNP0287952
609	D	-7.7	CNP0006605
610	D	-7.6	CNP0121052
611	D	-6.4	CNP0318509
612	D	-8.6	CNP0008435
613	D	-	CNP0161023
614	D	-8	CNP0089937
615	D	-7.9	CNP0217636
616	D	-6.4	CNP0125267
617	D	-7.7	CNP0396213
618	D	-8.6	CNP0383856
619	D	-6.7	CNP0285307
620	D	-7.3	CNP0225578
621	D	-6.7	CNP0321140
622	D	-6.7	CNP0138771
623	D	-7.3	CNP0290313
624	D	-6.5	CNP0220965
625	D	-6.5	CNP0360468
626	D	-8.2	CNP0195099
627	D	-7.4	CNP0145649
628	D	-7.2	CNP0140144
629	D	-7.2	CNP0006624
630	D	-6.2	CNP0104489
631	D	-6.6	CNP0128425
632	D	-6.5	CNP0128734
633	D	-6.5	CNP0382493
634	D	-7.4	CNP0409586
635	D	-	CNP0421741
636	D	-6.9	CNP0304287
637	D	-7.7	CNP0006630
638	D	-7.2	CNP0350404
639	D	-7.4	CNP0310792
640	D	-7	CNP0242364
641	D	-8.2	CNP0123056
642	D	-6.2	CNP0256611
643	D	-6.3	CNP0165997
644	D	-6.9	CNP0222315
645	D	-7.7	CNP0162517
646	D	-4.2	CNP0112376
647	D	-6.2	CNP0237412
648	D	-8	CNP0103109
649	D	-7.7	CNP0047866
650	D	-9.8	CNP0287338
651	D	-7.8	CNP0215063
652	D	-6.5	CNP0331030
653	D	-8.3	CNP0279714
654	D	-7	CNP0180067
655	D	-7.5	CNP0258771
656	D	-6.8	CNP0254486
657	D	-7.5	CNP0141860

658	D	-6.3	CNP0231090
659	D	-	CNP0150400
660	D	-6.4	CNP0174551
661	D	-7.5	CNP0243281
662	D	-6.7	CNP0303227
663	D	-7.7	CNP0420004
664	D	-6.4	CNP0232929
665	D	-6.3	CNP0326868
666	D	-6.3	CNP0213431
667	D	-6.4	CNP0219554
668	D	-7.2	CNP0177471
669	D	-7.4	CNP0272478
670	D	-7.3	CNP0013614
671	D	-7.3	CNP0203386
672	D	-7.2	CNP0183156
673	D	-7.2	CNP0176900
674	D	-8.4	CNP0092945
675	D	-8.1	CNP0162439
676	D	-9	CNP0098713
677	D	-8.7	CNP0209145
678	D	-7.8	CNP0331430
679	D	-8.5	CNP0097239
680	D	-8.1	CNP0111223
681	D	-7.9	CNP0211605
682	D	-8.3	CNP0008073
683	D	-8.4	CNP0277183
684	D	-8.3	CNP0239919
685	D	-8.6	CNP0179320
686	D	-7.7	CNP0219805
687	D	-8.6	CNP0228850
688	D	-7.9	CNP0421608
689	D	-9	CNP0087665
690	D	-8.8	CNP0401021
691	D	-7.8	CNP0188928
692	D	-7	CNP0275174
693	D	-8.9	CNP0294045
694	D	-6.8	CNP0415932
695	D	-6.9	CNP0319583
696	D	-9.1	CNP0266195
697	D	-8.1	CNP0156372
698	D	-7.6	CNP0340133
699	D	-8.2	CNP0255011
700	D	-9.3	CNP0013809
701	D	-8	CNP0308142
702	D	-8.1	CNP0006627
703	D	-8.1	CNP0288954
704	D	-7.6	CNP0202328
705	D	-7.9	CNP0008065
706	D	-7.7	CNP0293288
707	D	-7.7	CNP0246922
708	D	-	CNP0345022
709	D	-	CNP0100933
710	D	-	CNP0312050
711	D	-6	CNP0078153
712	D	-5.7	CNP0251793

713	D	-6.8	CNP0098529
714	D	-6.8	CNP0380844
715	D	-6.9	CNP0117020
716	D	-5.8	CNP0094081
717	D	-6	CNP0211370
718	D	-6	CNP0095711
719	D	-6.3	CNP0233323
720	D	-6.2	CNP0407204
721	D	-6.4	CNP0020760
722	D	-6	CNP0188798
723	D	-6.5	CNP0164590
724	D	-6	CNP0198465
725	D	-6.4	CNP0235799
726	D	-6.4	CNP0288614
727	D	-5.8	CNP0353229
728	D	-6.8	CNP0152723
729	D	-5.6	CNP0350481
730	D	-6	CNP0264351
731	D	-6.4	CNP0133008
732	D	-5.3	CNP0209926
733	D	-7.9	CNP0359638
734	D	-	CNP0188565
735	D	-3.1	CNP0421441
736	D	-6.8	CNP0357455
737	D	-5.7	CNP0337140
738	D	-5.2	CNP0174132
739	D	-4.9	CNP0290833
740	D	-5	CNP0277651
741	D	-4.9	CNP0242007
742	D	-6.8	CNP0093293
743	D	-5.3	CNP0224371
744	D	-7.2	CNP0230156
745	D	-6	CNP0129507
746	D	-5.3	CNP0257591
747	D	-5.4	CNP0202955
748	D	-6.4	CNP0263492
749	D	-5.9	CNP0100479
750	D	-5.7	CNP0358090
751	D	-5.5	CNP0048311
752	D	-1.5	CNP0408606
753	D	-4.8	CNP0148319
754	D	-3.7	CNP0421128
755	D	-6.6	CNP0096712
756	D	-5.6	CNP0126292
757	D	-6	CNP0350416
758	D	-7	CNP0147293
759	D	-0.7	CNP0351835
760	D	-4.9	CNP0211181
761	D	-6.1	CNP0412134
762	D	11.7	CNP0416362
763	D	-7	CNP0128652
764	D	-7.5	CNP0183689
765	D	-5.7	CNP0286882
766	D	-5.8	CNP0208611
767	D	-6.2	CNP0010478

768	D	-5.4	CNP0189327
769	D	-5.9	CNP0206774
770	D	-5.6	CNP0367988
771	D	151.9	CNP0097719
772	D	-6.3	CNP0420988
773	D	-5.7	CNP0188520
774	D	-7.1	CNP0422251
775	D	-6	CNP0196129
776	D	-5.3	CNP0111002
777	D	-5.4	CNP0233431
778	D	-5.7	CNP0310177
779	D	-5.8	CNP0179107
780	D	-6.6	CNP0116772
781	D	-5.3	CNP0324861
782	D	-5.1	CNP0251259
783	D	-5.6	CNP0305477
784	D	-3	CNP0008894
785	D	-5.2	CNP0393106
786	D	-6.3	CNP0101955
787	D	-5.4	CNP0290658
788	D	-5.1	CNP0291361
789	D	-5.4	CNP0346843
790	D	-6.4	CNP0060129
791	D	-5.1	CNP0302480
792	D	-5	CNP0065724
793	D	-	CNP0422881
794	D	-5.4	CNP0419134
795	D	-6.2	CNP0341922
796	D	-6.5	CNP0190291
797	D	-6.5	CNP0174836
798	D	-6.7	CNP0151500
799	D	-7.4	CNP0362893
800	D	-4.5	CNP0421418
801	D	-6.5	CNP0200161
802	D	-6.6	CNP0088687
803	D	-5.8	CNP0372438
804	D	-6	CNP0281556
805	D	-2.3	CNP0303682
806	D	-5.7	CNP0307704
807	D	-6.4	CNP0355612
808	D	-5.3	CNP0320938
809	D	-6.1	CNP0006733
810	D	-6.5	CNP0166643
811	D	-5.3	CNP0006596
812	D	-5.1	CNP0156976
813	D	-7.8	CNP0106576
814	D	-5.6	CNP0311941
815	D	-5.8	CNP0270182
816	D	-5.2	CNP0403240
817	D	-5.7	CNP0298394
818	D	-5.2	CNP0285684
819	D	-5.5	CNP0213107
820	D	-5	CNP0117286
821	D	-5.9	CNP0287913
822	D	-5.9	CNP0008063

823	D	-7.3	CNP0331041
824	D	-6.4	CNP0341794
825	D	-5.5	CNP0069439
826	D	-5.3	CNP0258515
827	D	-6.3	CNP0339361
828	D	-5.4	CNP0353773
829	D	-5.2	CNP0337562
830	D	-6.3	CNP0292947
831	D	3.6	CNP0089181
832	D	-5.7	CNP0180352
833	D	-6.2	CNP0225992
834	D	-5.4	CNP0228818
835	D	-6.8	CNP0104483
836	D	-6.5	CNP0155982
837	D	-6.1	CNP0159559
838	D	-6.1	CNP0237353
839	D	-6.6	CNP0282699
840	D	-6	CNP0205485
841	D	-6.6	CNP0192038
842	D	-5.9	CNP0230021
843	D	5.8	CNP0197136
844	D	-6	CNP0343768
845	D	-	CNP0091779
846	D	-6.3	CNP0134702
847	D	-6	CNP0047690
848	D	-5.9	CNP0350702
849	D	-6.2	CNP0255461
850	D	-5.6	CNP0142076
851	D	-5.9	CNP0381853
852	D	-5.1	CNP0095054
853	D	-6.1	CNP0354464
854	D	-5.5	CNP0238456
855	D	3.3	CNP0101438
856	D	-5.1	CNP0272858
857	D	80.1	CNP0225822
858	D	-7.7	CNP0046058
859	D	-6.1	CNP0101076
860	D	-5	CNP0043199
861	D	-5.9	CNP0023322
862	D	78.5	CNP0102200
863	D	-6.8	CNP0022622
864	D	-6.6	CNP0373950
865	D	-6	CNP0422122
866	D	-5.4	CNP0244633
867	D	-7.2	CNP0331197
868	D	-6.2	CNP0157537
869	D	-7.2	CNP0371058
870	D	-6.4	CNP0268083
871	D	-7.5	CNP0113573
872	D	-0.9	CNP0421365
873	D	-6	CNP0326562
874	D	-5.9	CNP0295735
875	D	-5.4	CNP0051679
876	D	-6.1	CNP0006748
877	D	-5.8	CNP0136451

878	D	-6.9	CNP0231351
879	D	-6.7	CNP0140866
880	D	-4.8	CNP0136427
881	D	-5.4	CNP0083934
882	D	-1.9	CNP0331341
883	D	-6.3	CNP0107041
884	D	-6.5	CNP0192511
885	D	-6.5	CNP0282489
886	D	-6.4	CNP0309264
887	D	-6	CNP0420066
888	D	-7.1	CNP0342837
889	D	-5.8	CNP0032043
890	D	-5.4	CNP0391314
891	D	-	CNP0101841
892	D	-5.8	CNP0315894
893	D	-6.8	CNP0421857
894	D	-6.4	CNP0337445
895	D	-2.7	CNP0305558
896	D	-5.7	CNP0075283
897	D	-6.4	CNP0000003
898	D	-5	CNP0337801
899	D	-5.8	CNP0157440
900	D	-5.9	CNP0260060
901	D	-7.3	CNP0076120
902	D	-7.3	CNP0167441
903	D	-7.6	CNP0237924
904	D	-6.7	CNP0090762
905	D	-6	CNP0180811
906	D	-7.3	CNP0092974
907	D	-6.1	CNP0350147
908	D	-6.3	CNP0099981
909	D	-8	CNP0167297
910	D	-6	CNP0275322
911	D	-6.9	CNP0100753
912	D	-6.5	CNP0088978
913	D	-6.1	CNP0376032
914	D	-7	CNP0318435
915	D	-7.5	CNP0287253
916	D	-6.8	CNP0335310
917	D	-6.1	CNP0351837
918	D	-7	CNP0176585
919	D	-7.1	CNP0238276
920	D	-6.5	CNP0212844
921	D	-6.7	CNP0297122
922	D	-6.7	CNP0372278
923	D	-6.2	CNP0316617
924	D	-6.1	CNP0367280
925	D	-5.2	CNP0343149
926	D	-6.1	CNP0243133
927	D	-7.3	CNP0165939
928	D	-6.6	CNP0088148
929	D	-6.2	CNP0260105
930	D	-6.3	CNP0341047
931	D	-	CNP0099306
932	D	-6.6	CNP0050463

933	D	-6.1	CNP0142684
934	D	-7.6	CNP0267268
935	D	-7.7	CNP0134978
936	D	-6.9	CNP0358092
937	D	-7.2	CNP0168009
938	D	-6.7	CNP0069988
939	D	-6.7	CNP0089477
940	D	-7.5	CNP0397153
941	D	-6.8	CNP0092238
942	D	-6.1	CNP0267605
943	D	-8.9	CNP0419934
944	D	-6.4	CNP0395221
945	D	-6.2	CNP0206918
946	D	-7.2	CNP0091718
947	D	-6.2	CNP0234360
948	D	-6.8	CNP0146300
949	D	-8	CNP0411216
950	D	-9.1	CNP0028979
951	D	-6.3	CNP0400525
952	D	-7.3	CNP0008402
953	D	-6.3	CNP0075567
954	D	-6.6	CNP0416521
955	D	-6.1	CNP0313702
956	D	-6.2	CNP0048518
957	D	-7.2	CNP0278283
958	D	-6.5	CNP0306839
959	D	-6	CNP0091701
960	D	-6.8	CNP0187662
961	D	-6.8	CNP0016052
962	D	-6.2	CNP0136160
963	D	-6	CNP0103020
964	D	-7.1	CNP0128249
965	D	-7.3	CNP0155654
966	D	-8.2	CNP0092533
967	D	-7.4	CNP0000011
968	D	-6.2	CNP0200446
969	D	-6.8	CNP0150205
970	D	-7.6	CNP0008097
971	D	-6.2	CNP0008071
972	D	-6.5	CNP0130013
973	D	-7.7	CNP0090195
974	D	-6.3	CNP0396044
975	D	-6.4	CNP0400455
976	D	-7.5	CNP0025404
977	D	-6.5	CNP0392070
978	D	-7.7	CNP0162358
979	D	-5.8	CNP0100124
980	D	-6.2	CNP0148625
981	D	-7.3	CNP0279272
982	D	-7.2	CNP0306385
983	D	-6.9	CNP0065791
984	D	-6.3	CNP0089784
985	D	-7.1	CNP0095038
986	D	-6.5	CNP0132397
987	D	-6.1	CNP0374953

988	D	-7.6	CNP0368176
989	D	-7.7	CNP0341211
990	D	-6.1	CNP0149341
991	D	-7.2	CNP0326177
992	D	-6.9	CNP0124653
993	D	-6.4	CNP0320374
994	D	-6.5	CNP0111814
995	D	-8	CNP0137147
996	D	-6.8	CNP0071038
997	D	-6.2	CNP0154364
998	D	-6.1	CNP0310666
999	D	-6.4	CNP0250428
1000	D	-6.4	CNP0296028
1001	D	-6.8	CNP0347789
1002	D	-7.3	CNP0090673
1003	D	-6.2	CNP0318132
1004	D	-6.7	CNP0420200
1005	D	-6.7	CNP0350555
1006	D	-6.1	CNP0156907
1007	D	-6.7	CNP0373966
1008	D	-6.4	CNP0008134
1009	D	-6.3	CNP0422593
1010	D	-7	CNP0060113
1011	D	-6.5	CNP0276991
1012	D	-7.2	CNP0013717
1013	D	-7.3	CNP0236381
1014	D	-7	CNP0254069
1015	D	-7.3	CNP0094054
1016	D	-6.3	CNP0204460
1017	D	-6.5	CNP0133342
1018	D	-6.5	CNP0125384
1019	D	-7.4	CNP0347421
1020	D	-7	CNP0420248
1021	D	-7.5	CNP0118659
1022	D	-6.4	CNP0393439
1023	D	-6.4	CNP0383512
1024	D	-6.2	CNP0323641
1025	D	-6.2	CNP0196247
1026	D	-6.2	CNP0244429
1027	D	-5.9	CNP0303456
1028	D	-5.7	CNP0398251
1029	D	-6.1	CNP0313758
1030	D	-6.2	CNP0099761
1031	D	-6.3	CNP0171699
1032	D	-6.8	CNP0387151
1033	D	-6.7	CNP0217275
1034	D	-6.3	CNP0162368
1035	D	-6.2	CNP0336240
1036	D	-6.8	CNP0334595
1037	D	-7.2	CNP0095034
1038	D	-7.4	CNP0300734
1039	D	-6.3	CNP0023694
1040	D	-7	CNP0028421
1041	D	-6.5	CNP0238308
1042	D	-7.7	CNP0182311

1043	D	-6	CNP0118222
1044	D	-6.6	CNP0324892
1045	D	-7.9	CNP0149003
1046	D	-6.2	CNP0400038
1047	D	-6.3	CNP0384074
1048	D	-6.3	CNP0263099
1049	D	-6.6	CNP0390499
1050	D	-7.2	CNP0142634
1051	D	-6.7	CNP0256140
1052	D	-6.4	CNP0222868
1053	D	-6.4	CNP0123489
1054	D	-6.9	CNP0080921
1055	D	-7.2	CNP0248106
1056	D	-6.7	CNP0353057
1057	D	-6.6	CNP0091185
1058	D	-6.7	CNP0393382
1059	D	-	CNP0101865
1060	D	-6.3	CNP0098035
1061	D	-7.1	CNP0008393
1062	D	-7.3	CNP0106204
1063	D	-6	CNP0243037
1064	D	-7	CNP0416238
1065	D	-7.4	CNP0095694
1066	D	-6.2	CNP0111764
1067	D	-7.1	CNP0279634
1068	D	-8.9	CNP0039463
1069	D	-7.9	CNP0243243
1070	D	-6	CNP0129127
1071	D	-6.9	CNP0310546
1072	D	-5.9	CNP0216374
1073	D	-7.1	CNP0359263
1074	D	-6.9	CNP0093372
1075	D	-6.3	CNP0422741
1076	D	-6.7	CNP0317013
1077	D	-7.6	CNP0148526
1078	D	-6.4	CNP0080919
1079	D	-	CNP0345547
1080	D	-6.7	CNP0114239
1081	D	-7.9	CNP0052958
1082	D	-6.7	CNP0204729
1083	D	-6.4	CNP0128259
1084	D	-6	CNP0075628
1085	D	-6.1	CNP0152694
1086	D	-7.1	CNP0008066
1087	D	-7.5	CNP0033877
1088	D	-6.7	CNP0087196
1089	D	-6.5	CNP0159173
1090	D	-6.4	CNP0242336
1091	D	-6.8	CNP0008061
1092	D	-7.5	CNP0341537
1093	D	-6.7	CNP0304859
1094	D	-7.6	CNP0169572
1095	D	-6.8	CNP0062538
1096	D	-6.3	CNP0141779
1097	D	-6.2	CNP0349798

1098	D	-	CNP0314491
1099	D	-7.4	CNP0320667
1100	D	-7.5	CNP0417948
1101	D	-7.4	CNP0337460
1102	D	-7.6	CNP0141882
1103	D	-7	CNP0312953
1104	D	-6.3	CNP0213714
1105	D	-6.1	CNP0307273
1106	D	-6.4	CNP0296547
1107	D	-6.9	CNP0365108
1108	D	-6.2	CNP0119979
1109	D	-6.6	CNP0298214
1110	D	-6.6	CNP0095699
1111	D	-8.2	CNP0092924
1112	D	-7.2	CNP0019483
1113	D	-7.3	CNP0284865
1114	D	-	CNP0094497
1115	D	-6.7	CNP0077438
1116	D	-6.4	CNP0139547
1117	D	-7.1	CNP0199139
1118	D	-7.3	CNP0357384
1119	D	-7.5	CNP0217477
1120	D	-5.9	CNP0131854
1121	D	-9	CNP0402556
1122	D	-6.5	CNP0186713
1123	D	-7	CNP0087476
1124	D	-6.4	CNP0008421
1125	D	-7.4	CNP0065069
1126	D	-6.7	CNP0262745
1127	D	-7	CNP0343648
1128	D	-6.5	CNP0186004
1129	D	-7.5	CNP0231286
1130	D	-6.8	CNP0229026
1131	D	-6.3	CNP0112624
1132	D	-8.1	CNP0447215
1133	D	-8.3	CNP0044563
1134	D	-6.1	CNP0008437
1135	D	-7.3	CNP0252381
1136	D	-6.8	CNP0219014
1137	D	-7.8	CNP0090178
1138	D	-6.5	CNP0089491
1139	D	-6.3	CNP0210017
1140	D	-6.2	CNP0166307
1141	D	-6.9	CNP0264139
1142	D	-7.7	CNP0013722
1143	D	-6.4	CNP0183579
1144	D	-	CNP0418536
1145	D	-6.3	CNP0006643
1146	D	-6.5	CNP0182346
1147	D	-6.9	CNP0101325
1148	D	-7.4	CNP0059869
1149	D	-6.6	CNP0180463
1150	D	-6.6	CNP0177079
1151	D	-5.8	CNP0379758
1152	D	-6.7	CNP0421099

1153	D	-7.1	CNP0261679
1154	D	-6	CNP0186565
1155	D	-5.7	CNP0253156
1156	D	-6.7	CNP0112215
1157	D	-7.3	CNP0244985
1158	D	-6.4	CNP0236253
1159	D	-6.3	CNP0213491
1160	D	-6.9	CNP0374472
1161	D	-6.6	CNP0166167
1162	D	-6.3	CNP0008060
1163	D	-7.5	CNP0041351
1164	D	-6.8	CNP0319955
1165	D	-7.2	CNP0380062
1166	D	-7.7	CNP0267335
1167	D	-7.5	CNP0075206
1168	D	-7.3	CNP0357389
1169	D	-7	CNP0346053
1170	D	-7.4	CNP0416772
1171	D	-6.3	CNP0312079
1172	D	-6.5	CNP0237561
1173	D	-5.9	CNP0253591
1174	D	-8.1	CNP0350586
1175	D	-6.1	CNP0046054
1176	D	-6.2	CNP0138878
1177	D	-6.7	CNP0364065
1178	D	-6.6	CNP0169391
1179	D	-7.2	CNP0097575
1180	D	-6.1	CNP0155908
1181	D	-6.4	CNP0173329
1182	D	-5.7	CNP0370167
1183	D	-6.5	CNP0302645
1184	D	-6.3	CNP0131571
1185	D	-7.3	CNP0106294
1186	D	-6.8	CNP0140930
1187	D	-6.2	CNP0265519
1188	D	-7.5	CNP0246784
1189	D	-6.2	CNP0023119
1190	D	-6.4	CNP0100484
1191	D	-6.2	CNP0293017
1192	D	-6.5	CNP0414102
1193	D	-5.8	CNP0263741
1194	D	-6.9	CNP0237149
1195	D	-6.1	CNP0257353
1196	D	-6.7	CNP0158405
1197	D	-7.2	CNP0385691
1198	D	-6.2	CNP0242090
1199	D	-7.1	CNP0147124
1200	D	-	CNP0091283
1201	D	-6.6	CNP0342286
1202	D	-7.4	CNP0046535
1203	D	-6.8	CNP0329869
1204	D	-6.5	CNP0156458
1205	D	-6.4	CNP0257962
1206	D	-5.5	CNP0319135
1207	D	-7.1	CNP0284970

1208	D	-6.9	CNP0193910
1209	D	-7	CNP0294654
1210	D	-8.3	CNP0350873
1211	D	-6.5	CNP0375014
1212	D	-6.3	CNP0024723
1213	D	-8.4	CNP0419618
1214	D	-6.2	CNP0234326
1215	D	-5.9	CNP0319358
1216	D	-6.6	CNP0335768
1217	D	-6.3	CNP0197528
1218	D	-7.1	CNP0008184
1219	D	-6.2	CNP0378655
1220	D	-5.7	CNP0151292
1221	D	-7.2	CNP0337973
1222	D	-6.4	CNP0280730
1223	D	-6.4	CNP0331809
1224	D	-6.5	CNP0173556
1225	D	-7.1	CNP0191680
1226	D	-5.7	CNP0103841
1227	D	-6	CNP0006604
1228	D	-6.7	CNP0293912
1229	D	-6.3	CNP0259538
1230	D	-8.1	CNP0415601
1231	D	-6	CNP0109128
1232	D	-6.5	CNP0196269
1233	D	-7	CNP0188440
1234	D	-7	CNP0421715
1235	D	-6.3	CNP0320181
1236	D	-6.4	CNP0152007
1237	D	-7.6	CNP0013535
1238	D	-5.4	CNP0417915
1239	D	-7	CNP0116520
1240	D	-6.2	CNP0128834
1241	D	-7.6	CNP0098733
1242	D	-6	CNP0033771
1243	D	-6.7	CNP0258595
1244	D	-6.8	CNP0148070
1245	D	-7.4	CNP0286773
1246	D	-7.6	CNP0270350
1247	D	-6.3	CNP0008247
1248	D	-6.9	CNP0138322
1249	D	-7.1	CNP0199391
1250	D	-7.2	CNP0309468
1251	D	-7.9	CNP0100397
1252	D	-8	CNP0088652
1253	D	-6.3	CNP0369718
1254	D	-6.3	CNP0235957
1255	D	-7.4	CNP0351489
1256	D	-7	CNP0313475
1257	D	-6.5	CNP0208621
1258	D	-6.4	CNP0288049
1259	D	-6.1	CNP0110048
1260	D	-5.8	CNP0009132
1261	D	-6.8	CNP0258192
1262	D	-6.4	CNP0302338

1263	D	-6.1	CNP0304175
1264	D	-7.5	CNP0008069
1265	D	-7.4	CNP0337295
1266	D	-6.9	CNP0351190
1267	D	-6.7	CNP0347151
1268	D	-6.6	CNP0037596
1269	D	-6.5	CNP0374709
1270	D	-6.5	CNP0420965
1271	D	-6.4	CNP0301849
1272	D	-6.5	CNP0229575
1273	D	-5.7	CNP0124693
1274	D	-6.1	CNP0219640
1275	D	-6.4	CNP0060033
1276	D	-5.3	CNP0146902
1277	D	-5.5	CNP0178618
1278	D	-4.9	CNP0210647
1279	D	-5.9	CNP0336077
1280	D	-5.6	CNP0192487
1281	D	-4.9	CNP0009274
1282	D	-7	CNP0173897
1283	D	-5.6	CNP0223720
1284	D	41.1	CNP0417551
1285	D	-5.3	CNP0259256
1286	D	-2.3	CNP0009296
1287	D	-	CNP0308572
1288	D	-6.4	CNP0098754
1289	D	-6	CNP0396977
1290	D	-6.8	CNP0013596
1291	D	-8.3	CNP0095705
1292	D	-6.2	CNP0118597
1293	D	-5.2	CNP0094922
1294	D	-6.6	CNP0213455
1295	D	-6.1	CNP0157334
1296	D	-7	CNP0031727
1297	D	-6.6	CNP0263399
1298	D	-6.3	CNP0280519
1299	D	-6.1	CNP0064136
1300	D	-6.8	CNP0263642
1301	D	-7.1	CNP0417037
1302	D	-5.3	CNP0095423
1303	D	-3.6	CNP0300567
1304	D	-5.8	CNP0378490
1305	D	-6.2	CNP0087887
1306	D	19.6	CNP0094524
1307	D	-5	CNP0044869
1308	D	-5.6	CNP0227401
1309	D	-6.2	CNP0045045
1310	D	-5.7	CNP0336497
1311	D	-5.9	CNP0214501
1312	D	-7.4	CNP0074192
1313	D	-5.5	CNP0138154
1314	D	-6.4	CNP0298101
1315	D	-5.9	CNP0306358
1316	D	-6.1	CNP0354767
1317	D	-5.7	CNP0310832

1318	D	-5.8	CNP0314198
1319	D	-5.4	CNP0144296
1320	D	-5.9	CNP0135818
1321	D	-5.2	CNP0277191
1322	D	-5.1	CNP0080920
1323	D	-6.3	CNP0109741
1324	D	-6.1	CNP0250701
1325	D	-6.3	CNP0035741
1326	D	-6	CNP0164275
1327	D	-6.1	CNP0167541
1328	D	-5.6	CNP0294735
1329	D	-5	CNP0100728
1330	D	-5.7	CNP0171717
1331	D	-6.7	CNP0286094
1332	D	-5	CNP0232231
1333	D	-5.7	CNP0140976
1334	D	-3.3	CNP0087977
1335	D	156.1	CNP0087975
1336	D	-6.1	CNP0238240
1337	D	-6.1	CNP0191094
1338	D	-6.4	CNP0108922
1339	D	-6.9	CNP0310452
1340	D	-5.6	CNP0357296
1341	D	-6.2	CNP0082225
1342	D	-5.7	CNP0191729
1343	D	-8.1	CNP0022825
1344	D	-5.4	CNP0255175
1345	D	-3.7	CNP0008059
1346	D	-5.7	CNP0372400
1347	D	-6	CNP0133001
1348	D	-5.1	CNP0122898
1349	D	57.7	CNP0051636
1350	D	-5.7	CNP0115741
1351	D	-5.5	CNP0327109
1352	D	-6.1	CNP0100503
1353	D	-	CNP0101078
1354	D	-6.5	CNP0104324
1355	D	-5.8	CNP0317418
1356	D	-0.7	CNP0157596
1357	D	-6.2	CNP0142870
1358	D	-5.8	CNP0231476
1359	D	-5.8	CNP0205397
1360	D	-5.6	CNP0350276
1361	D	-5.3	CNP0243026
1362	D	-5.7	CNP0263534
1363	D	-6.7	CNP0294166
1364	D	-5.5	CNP0091512
1365	D	-6.3	CNP0401669
1366	D	-6.7	CNP0314618
1367	D	40.8	CNP0344946
1368	D	-5.8	CNP0244586
1369	D	-6.2	CNP0242163
1370	D	-5.9	CNP0199802
1371	D	-	CNP0098133
1372	D	-3.5	CNP0271747

1373	D	-5.2	CNP0318764
1374	D	-6.1	CNP0165884
1375	D	-5.3	CNP0229546
1376	D	-5.9	CNP0190665
1377	D	-5.9	CNP0168286
1378	D	-5.5	CNP0187734
1379	D	-7	CNP0042846
1380	D	-6.1	CNP0256877
1381	D	-6.9	CNP0242545
1382	D	-6.7	CNP0294521
1383	D	-5.3	CNP0234064
1384	D	-	CNP0317095
1385	D	5.1	CNP0309810
1386	D	-6.2	CNP0026547
1387	D	-2.2	CNP0418781
1388	D	-6.1	CNP0227135
1389	D	-7	CNP0378921
1390	D	-6.2	CNP0053580
1391	D	-4.4	CNP0267370
1392	D	-6.9	CNP0068956
1393	D	-5.8	CNP0330647
1394	D	-5.6	CNP0008082
1395	D	-5.9	CNP0163668
1396	D	-6.2	CNP0261116
1397	D	-	CNP0102038
1398	D	-6.2	CNP0412486
1399	D	-	CNP0093481
1400	D	-5.4	CNP0202160
1401	D	107.9	CNP0129864
1402	D	3.3	CNP0401820
1403	D	-1.9	CNP0361934
1404	D	33	CNP0146736
1405	D	244.9	CNP0263617
1406	D	-5.5	CNP0359732
1407	D	-5.8	CNP0132410
1408	D	-5.4	CNP0289791
1409	D	-4.7	CNP0008068
1410	D	-6.1	CNP0220295
1411	D	-5.4	CNP0353630
1412	D	-5.7	CNP0087912
1413	D	-6.3	CNP0407238
1414	D	-6	CNP0304360
1415	D	-6.3	CNP0192731
1416	D	-6.7	CNP0263034
1417	D	-5.9	CNP0292296
1418	D	9.4	CNP0013810
1419	D	-4.8	CNP0093853
1420	D	-5.6	CNP0241417
1421	D	-6.3	CNP0301229
1422	D	-6.1	CNP0313282
1423	D	-5	CNP0266028
1424	D	-6.3	CNP0171584
1425	D	-5.1	CNP0343110
1426	D	-6.1	CNP0335064
1427	D	-5.6	CNP0122778

1428	D	-6.1	CNP0134879
1429	D	-5.5	CNP0257069
1430	D	-6.4	CNP0080912
1431	D	-5.8	CNP0349903
1432	D	-6	CNP0303570
1433	D	-5.7	CNP0153091
1434	D	-4.8	CNP0288627
1435	D	-6.2	CNP0259797
1436	D	1	CNP0282879
1437	D	-5.4	CNP0352068
1438	D	-7	CNP0081089
1439	D	-0.4	CNP0147503
1440	D	-4.7	CNP0262901
1441	D	-5.9	CNP0309850
1442	D	-5.4	CNP0217676
1443	D	-7.9	CNP0304349
1444	D	-5.9	CNP0140258
1445	D	-5.9	CNP0274382
1446	D	-5.9	CNP0278196
1447	D	-5.5	CNP0245347
1448	D	-5	CNP0148860
1449	D	-6	CNP0280021
1450	D	-5.4	CNP0324201
1451	D	-4.7	CNP0398695
1452	D	-5.8	CNP0212920
1453	D	36.7	CNP0418893
1454	D	-6.3	CNP0404234
1455	D	-5.3	CNP0302613
1456	D	-6.2	CNP0219577
1457	D	-	CNP0272254
1458	D	-4.7	CNP0057659
1459	D	-	CNP0423545
1460	D	-5.6	CNP0138295
1461	D	-4.6	CNP0213677
1462	D	-4.7	CNP0351566
1463	D	-6.1	CNP0133651
1464	D	-5.9	CNP0321086
1465	D	-5.1	CNP0343471
1466	D	-4.7	CNP0097543
1467	D	-6.4	CNP0256437
1468	D	-5.1	CNP0050086
1469	D	-6.6	CNP0287706
1470	D	45.8	CNP0365191
1471	D	-6.3	CNP0263958
1472	D	-1.2	CNP0208250
1473	D	-5.5	CNP0128659
1474	D	-5.5	CNP0245369
1475	D	-6.1	CNP0238378
1476	D	-5.5	CNP0351457
1477	D	-5.3	CNP0271840
1478	D	-4.9	CNP0095070
1479	D	-4.9	CNP0098989
1480	D	-4.5	CNP0167819
1481	D	-3.5	CNP0170015
1482	D	-5.2	CNP0355287

1483	D	-5.8	CNP0213748
1484	D	-5.8	CNP0101780
1485	D	-5.3	CNP0092278
1486	D	-5	CNP0351469
1487	D	2.1	CNP0180273
1488	D	-5.7	CNP0101379
1489	D	-4.4	CNP0227758
1490	D	-5.4	CNP0195097
1491	D	-5.1	CNP0095447
1492	D	-6.9	CNP0218489
1493	D	-4.9	CNP0309523
1494	D	-3.9	CNP0345147
1495	D	-4.4	CNP0077209
1496	D	-5.4	CNP0351744
1497	D	-6.2	CNP0202415
1498	D	-6.4	CNP0095108
1499	D	-5.9	CNP0112735
1500	D	-6.6	CNP0117010
1501	D	-5.7	CNP0113913
1502	D	-6.4	CNP0351780
1503	D	-6.1	CNP0301490
1504	D	-7.2	CNP0343442
1505	D	-7.3	CNP0096175
1506	D	-6.7	CNP0178195
1507	D	-5.9	CNP0134149
1508	D	-7	CNP0096193
1509	D	-7.3	CNP0163084
1510	D	-5	CNP0237152
1511	D	-7.3	CNP0094693
1512	D	-6.5	CNP0276149
1513	D	-7.7	CNP0137361
1514	D	-7.4	CNP0400334
1515	D	-6.5	CNP0107362
1516	D	-6.3	CNP0216412
1517	D	-5.7	CNP0331976
1518	D	-8	CNP0147076
1519	D	-6.9	CNP0191938
1520	D	-5.3	CNP0119580
1521	D	-6.9	CNP0422972
1522	D	-6.9	CNP0351743
1523	D	-7.5	CNP0283292
1524	D	-6.9	CNP0088168
1525	D	-7.8	CNP0188551
1526	D	-7.9	CNP0264927
1527	D	-7.7	CNP0210034
1528	D	-7	CNP0378738
1529	D	-7.1	CNP0329360
1530	D	-6.7	CNP0302453
1531	D	-8	CNP0340004
1532	D	-7.6	CNP0113553
1533	D	-6.6	CNP0105648
1534	D	-6	CNP0146405
1535	D	-7.2	CNP0079014
1536	D	-7.9	CNP0247173
1537	D	-6.2	CNP0182774

1538	D	-8.7	CNP0182670
1539	D	-6.3	CNP0227996
1540	D	-8.1	CNP0357456
1541	D	-6.6	CNP0311577
1542	D	-6.5	CNP0194820
1543	D	-6.5	CNP0412469
1544	D	-7.6	CNP0266033
1545	D	-6.4	CNP0184707
1546	D	-6.3	CNP0387376
1547	D	-6.9	CNP0306044
1548	D	-7.6	CNP0067683
1549	D	-7.2	CNP0297534
1550	D	-7.3	CNP0200030
1551	D	-7.1	CNP0219678
1552	D	-6.3	CNP0337637
1553	D	-6.1	CNP0105133
1554	D	-6.1	CNP0081085
1555	D	-5.7	CNP0354640
1556	D	-7.8	CNP0201401
1557	D	-6.5	CNP0113963
1558	D	-7.4	CNP0355202
1559	D	-6.4	CNP0184823
1560	D	-7	CNP0255347
1561	D	-6.2	CNP0154070
1562	D	-6.2	CNP0106510
1563	D	-6.2	CNP0095393
1564	D	-6.4	CNP0171144
1565	D	-6.2	CNP0112695
1566	D	-8.7	CNP0144788
1567	D	-6.3	CNP0144047
1568	D	-6.3	CNP0314335
1569	D	-6.4	CNP0088210
1570	D	-6.2	CNP0288711
1571	D	-8.3	CNP0314576
1572	D	-7.8	CNP0079015
1573	D	-7.8	CNP0239225
1574	D	-6.4	CNP0301887
1575	D	-6.8	CNP0118280
1576	D	-7	CNP0342229
1577	D	-6.5	CNP0234106
1578	D	-6.3	CNP0234086
1579	D	-6.2	CNP0158765
1580	D	-6.3	CNP0341181
1581	D	-6.1	CNP0292661
1582	D	-6.3	CNP0332997
1583	D	-6.4	CNP0102719
1584	D	-8.4	CNP0290931
1585	D	-6.5	CNP0108304
1586	D	-6.9	CNP0077393
1587	D	-6.4	CNP0183877
1588	D	-7.9	CNP0049329
1589	D	-6.3	CNP0311525
1590	D	-6	CNP0298647
1591	D	-6.1	CNP0248507
1592	D	-6.9	CNP0179081

1593	D	-7.9	CNP0182157
1594	D	-7	CNP0110881
1595	D	-7.2	CNP0027223
1596	D	-6	CNP0047791
1597	D	-6.3	CNP0180400
1598	D	-6.2	CNP0130219
1599	D	-7.2	CNP0149858
1600	D	-6.4	CNP0324122
1601	D	-5.5	CNP0309706
1602	D	-5.4	CNP0190857
1603	D	-7.4	CNP0308758
1604	D	-6.5	CNP0118431
1605	D	-5.9	CNP0394979
1606	D	-5.9	CNP0411380
1607	D	-6.1	CNP0306994
1608	D	-5.2	CNP0411218
1609	D	-5.5	CNP0288342
1610	D	-5.4	CNP0361982
1611	D	-6.4	CNP0354279
1612	D	-6.3	CNP0384338
1613	D	-5.4	CNP0151839
1614	D	-5.6	CNP0402470
1615	D	-6.3	CNP0235884
1616	D	-5.9	CNP0288228
1617	D	-5.5	CNP0333465
1618	D	-6	CNP0106269
1619	D	-5.5	CNP0328200
1620	D	-5.3	CNP0146917
1621	D	-5.2	CNP0356974
1622	D	-4.9	CNP0350474
1623	D	-6	CNP0161525
1624	D	-5.6	CNP0175114
1625	D	-5.5	CNP0239888
1626	D	-6	CNP0191485
1627	D	-5.8	CNP0267362
1628	D	-5.6	CNP0291216
1629	D	-6.3	CNP0259113
1630	D	-6	CNP0313761
1631	D	-4.8	CNP0224533
1632	D	-6.4	CNP0153433
1633	D	-5.6	CNP0212834
1634	D	-6.2	CNP0285540
1635	D	-5.8	CNP0227147
1636	D	-5.2	CNP0269863
1637	D	-5.7	CNP0097805
1638	D	-6.5	CNP0106548
1639	D	-6.3	CNP0389659
1640	D	-5.1	CNP0261418
1641	D	-6	CNP0230900
1642	D	-6.4	CNP0076516
1643	D	-5.5	CNP0306661
1644	D	-5.7	CNP0203670
1645	D	-5.2	CNP0171254
1646	D	-6.2	CNP0157746
1647	D	-6	CNP0292226

1648	D	-5.8	CNP0139266
1649	D	-5.7	CNP0285436
1650	D	-5.4	CNP0414508
1651	D	-6	CNP0017351
1652	D	-6.1	CNP0161172
1653	D	-6.2	CNP0194993
1654	D	-5.3	CNP0304012
1655	D	-5.9	CNP0113295
1656	D	-5.9	CNP0142216
1657	D	-6.1	CNP0172548
1658	D	-5.3	CNP0227466
1659	D	-6.4	CNP0251864
1660	D	-5.4	CNP0162363
1661	D	-5.7	CNP0296919
1662	D	-5.3	CNP0327287
1663	D	-6.7	CNP0392138
1664	D	-5.8	CNP0169356
1665	D	-5.9	CNP0201735
1666	D	-6.4	CNP0198340
1667	D	-5.1	CNP0323699
1668	D	-5.9	CNP0180200
1669	D	-5.5	CNP0354646
1670	D	-6.1	CNP0139091
1671	D	-5.8	CNP0311650
1672	D	-6.1	CNP0364486
1673	D	-7.8	CNP0340346
1674	D	-6.1	CNP0263582
1675	D	-5	CNP0280571
1676	D	-5.7	CNP0306073
1677	D	-5.8	CNP0151165
1678	D	-6.2	CNP0221490
1679	D	-5.8	CNP0325899
1680	D	-6.2	CNP0118228
1681	D	-5.7	CNP0300662
1682	D	-5.6	CNP0324109
1683	D	-5.7	CNP0346292
1684	D	-5.8	CNP0214831
1685	D	-7.7	CNP0187203
1686	D	-5.5	CNP0289413
1687	D	-5.3	CNP0252617
1688	D	-6.2	CNP0335083
1689	D	-5.8	CNP0355146
1690	D	-4.5	CNP0237018
1691	D	-6.7	CNP0107887
1692	D	-5.8	CNP0255706
1693	D	-5.4	CNP0365427
1694	D	-6.9	CNP0199204
1695	D	-5.9	CNP0357437
1696	D	-5.7	CNP0151193
1697	D	-5.5	CNP0169496
1698	D	-5.4	CNP0248348
1699	D	-5.7	CNP0251715
1700	D	-6.3	CNP0279645
1701	D	-6.4	CNP0166107
1702	D	-5.7	CNP0165925

1703	D	-6.5	CNP0183712
1704	D	-5.4	CNP0419667
1705	D	-5.6	CNP0226211
1706	D	-5.8	CNP0126790
1707	D	-5.6	CNP0190667
1708	D	-5.8	CNP0310358
1709	D	-5.6	CNP0182388
1710	D	-7	CNP0221448
1711	D	-6.4	CNP0283600
1712	D	-5.3	CNP0164371
1713	D	-5.5	CNP0257415
1714	D	-6	CNP0231721
1715	D	-5.1	CNP0141998
1716	D	-6.8	CNP0323188
1717	D	-5.8	CNP0229155
1718	D	-6.8	CNP0197465
1719	D	-7	CNP0333939
1720	D	-5.7	CNP0125310
1721	D	-5.5	CNP0196160
1722	D	-5.5	CNP0235907
1723	D	-6.1	CNP0268692
1724	D	-4.9	CNP0180817
1725	D	-5.6	CNP0191629
1726	D	-5.5	CNP0291153
1727	D	-6.3	CNP0228076
1728	D	-5.7	CNP0168565
1729	D	-5.5	CNP0341582
1730	D	-5.1	CNP0334573
1731	D	-6.5	CNP0356087
1732	D	-6.8	CNP0351668
1733	D	-5.2	CNP0299454
1734	D	-5.6	CNP0200409
1735	D	-5.6	CNP0175999
1736	D	-5.7	CNP0181721
1737	D	-5.8	CNP0192125
1738	D	-5.4	CNP0155046
1739	D	-7.1	CNP0161453
1740	D	-5.8	CNP0331090
1741	D	-5.3	CNP0225911
1742	D	-5.6	CNP0353285
1743	D	-5.1	CNP0311729
1744	D	-6.2	CNP0214195
1745	D	-5.6	CNP0354530
1746	D	-6.1	CNP0178668
1747	D	-5.5	CNP0191160
1748	D	-6.8	CNP0363051
1749	D	-5.6	CNP0226658
1750	D	-6	CNP0250696
1751	D	-6.5	CNP0277958
1752	D	-6.4	CNP0287714
1753	D	-6.2	CNP0202606
1754	D	-5.3	CNP0108797
1755	D	-5.3	CNP0246165
1756	D	-7	CNP0321077
1757	D	-6	CNP0353291

1758	D	-5.4	CNP0301488
1759	D	-6.1	CNP0256332
1760	D	-5.7	CNP0294706
1761	D	-5.3	CNP0314896
1762	D	-5.4	CNP0327714
1763	D	-6.3	CNP0172827
1764	D	-5.6	CNP0123296
1765	D	-6.6	CNP0338965
1766	D	-5.3	CNP0285625
1767	D	-5.5	CNP0331652
1768	D	-5.5	CNP0264940
1769	D	-5.5	CNP0112196
1770	D	-5.6	CNP0401814
1771	D	-5.8	CNP0171650
1772	D	-5.7	CNP0297047
1773	D	-5.9	CNP0243443
1774	D	-6	CNP0278552
1775	D	-5.7	CNP0351760
1776	D	-5.4	CNP0194273
1777	D	-5.3	CNP0240762
1778	D	-5.8	CNP0205288
1779	D	-5	CNP0418121
1780	D	-5.4	CNP0265892
1781	D	-5.4	CNP0152445
1782	D	-5.4	CNP0245251
1783	D	-6.3	CNP0351077
1784	D	-5.7	CNP0329735
1785	D	-5.5	CNP0314571
1786	D	-7.1	CNP0350321
1787	D	-5.5	CNP0111646
1788	D	-6.2	CNP0212518
1789	D	-5.6	CNP0211862
1790	D	-6.8	CNP0372024
1791	D	-5.6	CNP0304018
1792	D	-5.6	CNP0192482
1793	D	-5.3	CNP0215616
1794	D	-5.4	CNP0355607
1795	D	-6.1	CNP0168367
1796	D	-5.8	CNP0010279
1797	D	-5.4	CNP0353693
1798	D	-5.8	CNP0137241
1799	D	-6	CNP0139478
1800	D	-5	CNP0325455
1801	D	-5.7	CNP0151995
1802	D	-5.5	CNP0246555
1803	D	-6.6	CNP0330930
1804	D	-5.7	CNP0133768
1805	D	-6.4	CNP0182394
1806	D	-5.2	CNP0333008
1807	D	-5.6	CNP0189466
1808	D	-6.2	CNP0307880
1809	D	-5.6	CNP0170811
1810	D	-5.3	CNP0145023
1811	D	-5.4	CNP0247288
1812	D	-5.6	CNP0192640

1813	D	-5.9	CNP0218001
1814	D	-5.6	CNP0337197
1815	D	-5.7	CNP0100116
1816	D	-6.8	CNP0382857
1817	D	-6.8	CNP0224222
1818	D	-5.7	CNP0304411
1819	D	-6.3	CNP0322743
1820	D	-5.9	CNP0185632
1821	D	-5.5	CNP0279577
1822	D	-5.4	CNP0224264
1823	D	-5.8	CNP0323373
1824	D	-6.8	CNP0076657
1825	D	-5.8	CNP0359622
1826	D	-5.5	CNP0271502
1827	D	-5.6	CNP0075632
1828	D	-6.2	CNP0199025
1829	D	-5.7	CNP0126370
1830	D	-5.4	CNP0151591
1831	D	-5.9	CNP0145199
1832	D	-6.4	CNP0146067
1833	D	-5.4	CNP0077177
1834	D	-5.6	CNP0170591
1835	D	-6.8	CNP0395770
1836	D	-5.7	CNP0310523
1837	D	-6.1	CNP0105753
1838	D	-5.6	CNP0208506
1839	D	-5.4	CNP0332208
1840	D	-5.7	CNP0193269
1841	D	-5.6	CNP0240897
1842	D	-6.7	CNP0104322
1843	D	-6.5	CNP0271835
1844	D	-5.5	CNP0250013
1845	D	-5.3	CNP0112926
1846	D	-5.7	CNP0264805
1847	D	-5.5	CNP0382236
1848	D	-5.5	CNP0328696
1849	D	-5.4	CNP0131900
1850	D	-5.8	CNP0408826
1851	D	-6.5	CNP0243041
1852	D	-5.9	CNP0196388
1853	D	-6.2	CNP0244395
1854	D	-6.2	CNP0153309
1855	D	-6.7	CNP0202672
1856	D	-6.1	CNP0318696
1857	D	-6.5	CNP0151248
1858	D	-6.9	CNP0120747
1859	D	-7.2	CNP0373965
1860	D	-7	CNP0395204
1861	D	-6.2	CNP0109483
1862	D	-6.9	CNP0354953
1863	D	-7.1	CNP0156081
1864	D	-7.1	CNP0351761
1865	D	-6.4	CNP0332920
1866	D	-6.5	CNP0281175
1867	D	-6.5	CNP0126118

1868	D	-6.6	CNP0343040
1869	D	-6.8	CNP0246923
1870	D	-6.8	CNP0183367
1871	D	-7.2	CNP0215582
1872	D	-6.5	CNP0142118
1873	D	-6.3	CNP0216228
1874	D	-6.3	CNP0214839
1875	D	-7	CNP0198783
1876	D	-6.3	CNP0211296
1877	D	-6.6	CNP0314005
1878	D	-6.5	CNP0180638
1879	D	-6.2	CNP0162853
1880	D	-5.7	CNP0257447
1881	D	-6.5	CNP0174138
1882	D	-6.4	CNP0186624
1883	D	-6.4	CNP0165804
1884	D	-6.1	CNP0239290
1885	D	-7.3	CNP0130897
1886	D	-7.6	CNP0300369
1887	D	-7.1	CNP0161155
1888	D	-6.9	CNP0355207
1889	D	-6.8	CNP0159352
1890	D	-6.3	CNP0339357
1891	D	-5.8	CNP0168068
1892	D	-6.2	CNP0411393
1893	D	-6.2	CNP0279137
1894	D	-6.5	CNP0161815
1895	D	-6.7	CNP0281359
1896	D	-6.4	CNP0274721
1897	D	-6.3	CNP0122160
1898	D	-6.5	CNP0261803
1899	D	-7	CNP0174843
1900	D	-6.5	CNP0412415
1901	D	-6.4	CNP0175439
1902	D	-7.2	CNP0351746
1903	D	-6.4	CNP0127424
1904	D	-6.4	CNP0103836
1905	D	-6	CNP0137156
1906	D	-6.5	CNP0211202
1907	D	-6.4	CNP0148469
1908	D	-6.5	CNP0171787
1909	D	-7.1	CNP0146230
1910	D	-6.6	CNP0270330
1911	D	-7.2	CNP0164308
1912	D	-7.2	CNP0132415
1913	D	-6.2	CNP0278335
1914	D	-6.3	CNP0123967
1915	D	-6.4	CNP0205678
1916	D	-6.5	CNP0305339
1917	D	-6.5	CNP0200423
1918	D	-6.2	CNP0080149
1919	D	-8	CNP0048261
1920	D	-6.8	CNP0158246
1921	D	-7.2	CNP0149022
1922	D	-6.3	CNP0142378

1923	D	-6.8	CNP0137662
1924	D	-5.5	CNP0379739
1925	D	-7.2	CNP0392190
1926	D	-6.7	CNP0392269
1927	D	-6.5	CNP0091610
1928	D	-7.4	CNP0126249
1929	D	-5.6	CNP0131042
1930	D	-7	CNP0157749
1931	D	-6.1	CNP0115565
1932	D	-7.2	CNP0356151
1933	D	-6.1	CNP0098123
1934	D	-6.3	CNP0200730
1935	D	-6.6	CNP0103632
1936	D	-7.4	CNP0272111
1937	D	-6.8	CNP0080931
1938	D	-6.1	CNP0266623
1939	D	-6.3	CNP0218500
1940	D	-5.9	CNP0111894
1941	D	-6.7	CNP0196967
1942	D	-6.2	CNP0133530
1943	D	-7.2	CNP0286712
1944	D	-5.5	CNP0285894
1945	D	-6.4	CNP0151684
1946	D	-7.1	CNP0208554
1947	D	-6.4	CNP0237024
1948	D	-6.3	CNP0174545
1949	D	-6.3	CNP0349610
1950	D	-6.5	CNP0105282
1951	D	-6.2	CNP0254544
1952	D	-6.9	CNP0141475
1953	D	-6.5	CNP0326474
1954	D	-7.1	CNP0079017
1955	D	-6	CNP0141519
1956	D	-6.3	CNP0220133
1957	D	-8	CNP0238890
1958	D	-6.1	CNP0061449
1959	D	-5.5	CNP0136402
1960	D	-6.2	CNP0352097
1961	D	-6.4	CNP0208007
1962	D	-6.5	CNP0188018
1963	D	-6	CNP0321226
1964	D	-7.1	CNP0148222
1965	D	-6.5	CNP0240205
1966	D	-6.2	CNP0417617
1967	D	-5.9	CNP0160725
1968	D	-6.2	CNP0320966
1969	D	-6.5	CNP0187750
1970	D	-6.5	CNP0141608
1971	D	-6	CNP0225654
1972	D	-6.3	CNP0163832
1973	D	-6.6	CNP0221540
1974	D	-6.4	CNP0299429
1975	D	-6.3	CNP0331006
1976	D	-6.3	CNP0114947
1977	D	-6.6	CNP0122166

1978	D	-6.6	CNP0150744
1979	D	-6.3	CNP0265835
1980	D	-6.5	CNP0252740
1981	D	-6.2	CNP0212583
1982	D	-6.6	CNP0372394
1983	D	-6.3	CNP0158342
1984	D	-6.6	CNP0207191
1985	D	-6.5	CNP0229850
1986	D	-6.8	CNP0292554
1987	D	-6.1	CNP0202387
1988	D	-5.7	CNP0364779
1989	D	-6.4	CNP0135646
1990	D	-6.7	CNP0197532
1991	D	-7.1	CNP0199855
1992	D	-6.2	CNP0329805
1993	D	-7	CNP0248036
1994	D	-7.7	CNP0342275
1995	D	-6.3	CNP0288063
1996	D	-6.9	CNP0377239
1997	D	-6.3	CNP0135948
1998	D	-6.1	CNP0114751
1999	D	-6.4	CNP0111683
2000	D	-6.8	CNP0204248
2001	D	-6.1	CNP0192526
2002	D	-6.1	CNP0354852
2003	D	-6.4	CNP0422955
2004	D	-6.3	CNP0125670
2005	D	-6.1	CNP0237349
2006	D	-6.2	CNP0105296
2007	D	-6.5	CNP0253334
2008	D	-6.6	CNP0252982
2009	D	-5.3	CNP0383560
2010	D	-6.5	CNP0130904
2011	D	-6.6	CNP0321276
2012	D	-6.4	CNP0182076
2013	D	-5.9	CNP0249879
2014	D	-6.5	CNP0120035
2015	D	-6.2	CNP0171115
2016	D	-6.6	CNP0199512
2017	D	-6.7	CNP0116115
2018	D	-5.9	CNP0354644
2019	D	-6.3	CNP0114229
2020	D	-6.6	CNP0255726
2021	D	-7.2	CNP0355011
2022	D	-6.3	CNP0076192
2023	D	-6.5	CNP0365883
2024	D	-6.1	CNP0234699
2025	D	-6.4	CNP0378427
2026	D	-6.1	CNP0321443
2027	D	-7.7	CNP0181976
2028	D	-7.1	CNP0304090
2029	D	-6.6	CNP0180280
2030	D	-6.9	CNP0153541
2031	D	-8.3	CNP0155740
2032	D	-6.8	CNP0080928

2033	D	-6.4	CNP0284160
2034	D	-6.6	CNP0230705
2035	D	-6.3	CNP0263056
2036	D	-6.3	CNP0175864
2037	D	-6.8	CNP0115254
2038	D	-6.5	CNP0292500
2039	D	-6.8	CNP0312517
2040	D	-6.3	CNP0294203
2041	D	-6.3	CNP0180706
2042	D	-6.4	CNP0093208
2043	D	-6.8	CNP0130645
2044	D	-6.3	CNP0269133
2045	D	-6.1	CNP0117952
2046	D	-5.8	CNP0059265
2047	D	-6.3	CNP0231320
2048	D	-8.1	CNP0183180
2049	D	-6.4	CNP0377623
2050	D	-6.9	CNP0194680
2051	D	-6.4	CNP0216466
2052	D	-6	CNP0210144
2053	D	-6.8	CNP0288191
2054	D	-6.4	CNP0281361
2055	D	-6.1	CNP0348752
2056	D	-6.6	CNP0297717
2057	D	-6.3	CNP0262423
2058	D	-6.2	CNP0166975
2059	D	-6.4	CNP0271155
2060	D	-6.6	CNP0309416
2061	D	-6.6	CNP0243364
2062	D	-6.6	CNP0374639
2063	D	-6.3	CNP0357434
2064	D	-6.8	CNP0202865
2065	D	-6.7	CNP0267003
2066	D	-5.8	CNP0233938
2067	D	-7	CNP0134525
2068	D	-6.1	CNP0356373
2069	D	-6.6	CNP0124831
2070	D	-6.2	CNP0122552
2071	D	-6.5	CNP0140382
2072	D	-6.8	CNP0232347
2073	D	-7.9	CNP0401680
2074	D	-6.3	CNP0212287
2075	D	-6.6	CNP0129822
2076	D	-6.5	CNP0149041
2077	D	-6.5	CNP0108915
2078	D	-6.7	CNP0229019
2079	D	-7.5	CNP0305276
2080	D	-6.5	CNP0313605
2081	D	-7.2	CNP0124237
2082	D	-6.5	CNP0308908
2083	D	-6.8	CNP0168846
2084	D	-6.6	CNP0224812
2085	D	-8.1	CNP0412420
2086	D	-6.3	CNP0277104
2087	D	-6.4	CNP0269430

2088	D	-6.3	CNP0342011
2089	D	-6.2	CNP0194341
2090	D	-6.5	CNP0367220
2091	D	-6.3	CNP0330694
2092	D	-6.4	CNP0342604
2093	D	-6.8	CNP0142159
2094	D	-7.1	CNP0282023
2095	D	-6.4	CNP0140985
2096	D	-5.6	CNP0151031
2097	D	-6.4	CNP0148527
2098	D	-6.6	CNP0277910
2099	D	-6.3	CNP0330872
2100	D	-6.2	CNP0255907
2101	D	-6.1	CNP0193799
2102	D	-7	CNP0144560
2103	D	-6.2	CNP0301073
2104	D	-6.4	CNP0374586
2105	D	-6.6	CNP0375467
2106	D	-6.9	CNP0318670
2107	D	-6.2	CNP0288492
2108	D	-6.2	CNP0249479
2109	D	-6.4	CNP0323363
2110	D	-6.7	CNP0136220
2111	D	-6.2	CNP0221067
2112	D	-6.8	CNP0103832
2113	D	-6.3	CNP0303069
2114	D	-6.6	CNP0204039
2115	D	-6.7	CNP0298805
2116	D	-6.2	CNP0125452
2117	D	-6.9	CNP0357016
2118	D	-6.4	CNP0282947
2119	D	-6.2	CNP0042181
2120	D	-6.2	CNP0092627
2121	D	-6.4	CNP0144213
2122	D	-7.1	CNP0355200
2123	D	-8.3	CNP0258991
2124	D	-6.2	CNP0286939
2125	D	-6.5	CNP0354817
2126	D	-6.4	CNP0075882
2127	D	-6.3	CNP0369937
2128	D	-6.1	CNP0353663
2129	D	-6.6	CNP0135697
2130	D	-7	CNP0080249
2131	D	-7.9	CNP0196298
2132	D	-6.3	CNP0298621
2133	D	-6.1	CNP0148112
2134	D	-6.2	CNP0309047
2135	D	-6.1	CNP0274375
2136	D	-6.3	CNP0273848
2137	D	-6.1	CNP0253857
2138	D	-6.2	CNP0176104
2139	D	-7.2	CNP0221883
2140	D	-6.5	CNP0320227
2141	D	-6.1	CNP0138634
2142	D	-6.1	CNP0354641

2143	D	-6.2	CNP0316629
2144	D	-6.9	CNP0365247
2145	D	-7.3	CNP0226307
2146	D	-6.5	CNP0190680
2147	D	-6.4	CNP0226088
2148	D	-6.4	CNP0306959
2149	D	-6.3	CNP0194135
2150	D	-6.5	CNP0154461
2151	D	-5.7	CNP0188022
2152	D	-6.8	CNP0388459
2153	D	-6	CNP0257014
2154	D	-6.4	CNP0359409
2155	D	-6.4	CNP0276114
2156	D	-6.3	CNP0270662
2157	D	-6.5	CNP0277917
2158	D	-6.6	CNP0259489
2159	D	-6.9	CNP0339906
2160	D	-6.5	CNP0312354
2161	D	-5.4	CNP0338215
2162	D	-6	CNP0330670
2163	D	-6.5	CNP0230375
2164	D	-5.8	CNP0119272
2165	D	-6.9	CNP0228197
2166	D	-5.9	CNP0316533
2167	D	-6.1	CNP0184533
2168	D	-6.7	CNP0279349
2169	D	-6.4	CNP0107167
2170	D	-6.3	CNP0336157
2171	D	-6.1	CNP0274499
2172	D	-6.2	CNP0169861
2173	D	-6.1	CNP0306155
2174	D	-6.4	CNP0133383
2175	D	-6.2	CNP0388547
2176	D	-7.5	CNP0324285
2177	D	-6.3	CNP0260434
2178	D	-6.1	CNP0233755
2179	D	-6.5	CNP0272552
2180	D	-6.6	CNP0201863
2181	D	-6	CNP0120508
2182	D	-6.4	CNP0418004
2183	D	-6.7	CNP0224726
2184	D	-6.1	CNP0203874
2185	D	-5.8	CNP0178572
2186	D	-5.8	CNP0132378
2187	D	-6.7	CNP0321391
2188	D	-6.3	CNP0171092
2189	D	-6.4	CNP0231501
2190	D	-6.5	CNP0369739
2191	D	-6.4	CNP0409338
2192	D	-6.6	CNP0025449
2193	D	-6.2	CNP0282240
2194	D	-6.2	CNP0278624

**Table S2**: Dataset of 76 natural AQs-related compounds from COCONUT [55] with docking score			
**S.No.**		**Binding Affinity (kcal/mol)**	**Coconut_id**
465	D	-9.8	CNP0000012
302	D	-9.5	CNP0097600
52	D	-9.4	CNP0229787
310	D	-9.4	CNP0019876
298	D	-9.2	CNP0129460
100	D	-9.2	CNP0309114
950	D	-9.1	CNP0028979
339	D	-9	CNP0080910
132	D	-9	CNP0316412
329	D	-8.9	CNP0140926
551	D	-8.9	CNP0113212
1068	D	-8.9	CNP0039463
335	D	-8.9	CNP0300624
534	D	-8.8	CNP0311311
199	D	-8.8	CNP0281894
482	D	-8.8	CNP0184132
677	D	-8.7	CNP0209145
1566	D	-8.7	CNP0144788
382	D	-8.7	CNP0061269
233	D	-8.7	CNP0181567
582	D	-8.7	CNP0006762
478	D	-8.6	CNP0388669
462	D	-8.6	CNP0311496
450	D	-8.6	CNP0113228
88	D	-8.6	CNP0339449
618	D	-8.6	CNP0383856
517	D	-8.5	CNP0378596
86	D	-8.5	CNP0304999
421	D	-8.5	CNP0356198
225	D	-8.5	CNP0224978
507	D	-8.4	CNP0379931
130	D	-8.4	CNP0197369
187	D	-8.4	CNP0118833
521	D	-8.4	CNP0125178
566	D	-8.4	CNP0117276
580	D	-8.4	CNP0166260
1584	D	-8.4	CNP0290931
1213	D	-8.4	CNP0419618
345	D	-8.3	CNP0199241
2031	D	-8.3	CNP0155740
653	D	-8.3	CNP0279714
1210	D	-8.3	CNP0350873
242	D	-8.3	CNP0166617
358	D	-8.2	CNP0333756
699	D	-8.2	CNP0255011
449	D	-8.2	CNP0323961
440	D	-8.2	CNP0395463
703	D	-8.1	CNP0288954

675	D	-8.1	CNP0162439
338	D	-8.1	CNP0363606
549	D	-8.1	CNP0025327
319	D	-8.1	CNP0139138
301	D	-8.1	CNP0110759
296	D	-8.1	CNP0295613
226	D	-8.1	CNP0176439
209	D	-8.1	CNP0474581
293	D	-8.1	CNP0115064
362	D	-8.1	CNP0267295
1540	D	-8.1	CNP0357456
203	D	-8.1	CNP0418451
1132	D	-8.1	CNP0447215
1343	D	-8.1	CNP0022825
65	D	-8.1	CNP0219593
68	D	-8.1	CNP0327787
701	D	-8	CNP0308142
177	D	-8	CNP0111804
333	D	-8	CNP0272431
497	D	-8	CNP0106464
141	D	-8	CNP0277276
995	D	-8	CNP0137147
1957	D	-8	CNP0238890
205	D	-8	CNP0282759
262	D	-8	CNP0230160
138	D	-8	CNP0130818
178	D	-8	CNP0138873
909	D	-8	CNP0167297
